# Differential Response of MYB Transcription Factor Gene Transcripts to Circadian Rhythm in Tea Plants (*Camellia sinensis*)

**DOI:** 10.3390/ijms25010657

**Published:** 2024-01-04

**Authors:** Zhihang Hu, Nan Zhang, Zhiyuan Qin, Jinwen Li, Ni Yang, Yi Chen, Jieyu Kong, Wei Luo, Aisheng Xiong, Jing Zhuang

**Affiliations:** 1 Tea Science Research Institute, College of Horticulture, Nanjing Agricultural University, Nanjing 210095, China; 2022204007@stu.njau.edu.cn (Z.H.); 2022804179@stu.njau.edu.cn (Z.Q.); 2020204040@stu.njau.edu.cn (J.L.); 2021204040@stu.njau.edu.cn (N.Y.); 2019204042@njau.edu.cn (Y.C.); 2021104085@stu.njau.edu.cn (J.K.); 2022104084@stu.njau.edu.cn (W.L.); 2 State Key Laboratory of Crop Genetics & Germplasm Enhancement and Utilization, Nanjing Agricultural University, Nanjing 210095, China; 2022104054@stu.njau.edu.cn

**Keywords:** *Camellia sinensis*, MYB, circadian rhythm, transcription factor, transcriptome analysis

## Abstract

The circadian clock refers to the formation of a certain rule in the long-term evolution of an organism, which is an invisible ‘clock’ in the body of an organism. As one of the largest TF families in higher plants, the MYB transcription factor is involved in plant growth and development. MYB is also inextricably correlated with the circadian rhythm. In this study, the transcriptome data of the tea plant ‘Baiyeyihao’ were measured at a photoperiod interval of 4 h (24 h). A total of 25,306 unigenes were obtained, including 14,615 unigenes that were annotated across 20 functional categories within the GO classification. Additionally, 10,443 single-gene clusters were annotated to 11 sublevels of metabolic pathways using KEGG. Based on the results of gene annotation and differential gene transcript analysis, 22 genes encoding MYB transcription factors were identified. The G10 group in the phylogenetic tree had 13 members, of which 5 were related to the circadian rhythm, accounting for 39%. The G1, G2, G8, G9, G15, G16, G18, G19, G20, G21 and G23 groups had no members associated with the circadian rhythm. Among the 22 differentially expressed MYB transcription factors, 3 members of LHY, RVE1 and RVE8 were core circadian rhythm genes belonging to the G10, G12 and G10 groups, respectively. Real-time fluorescence quantitative PCR was used to detect and validate the expression of the gene transcripts encoding MYB transcription factors associated with the circadian rhythm.

## 1. Introduction

The circadian rhythm is an endogenous timing mechanism that operates on a 24 h cycle, reflecting the relative length of day and night [1]. The input, central oscillator and output pathways are the three components that constitute the circadian rhythm system in higher plants. Among them, the central oscillator, which is the central element of the biological clock system, is essential for controlling plant growth [2,3]. Previous studies have identified core clock genes, such as the *CIRCADIAN CLOCK ASSOCIATED 1* (*CCA1*), *PSEUDO RESPONSE REGULATOR* (*PRR*) family, *TIMING OF CAB EXPRESSION 1* (*TOC1*), *GIGANTEA* (*GI*), *REVEILLEs* (*RVEs*), *NIGHT LIGHT-INDUCIBLE AND CLOCK-REGULATED* (*LNK*) and *EARLY FLOWERING* (*ELF*) genes [4]. At present, research related to the circadian rhythm mainly focuses on *Arabidopsis*, rice, tomato, etc. [5,6,7]. Huang et al. investigated photosynthetic efficiency through the circadian clock network under different light intensities in tomatoes [7]. The appearance of mutations in EID1 and LNK2 during domestication delayed both the phase and period of its circadian rhythm [6]. Most clock genes in *Arabidopsis* have homologous genes in rice [8]. *OsELF3* promotes flowering by repressing *Ghd7* at the transcriptional level and maintains circadian accumulation by interacting with *HAF1* under long-day conditions [9]. The photoperiodic control of flowering time and photoregulation of growth are two examples of features that can be altered by clock gene mutations [10,11]. When comparing the phenotypes of wild-type and mutant plants with clock-changing functions, it can be found that different physiological characteristics (total CO_2_ assimilation rate, sugar status and photosynthetic utilization efficiency) are affected by the circadian rhythm [12,13]. The expression profiles of genes related to the circadian rhythm show different patterns in the photoperiods of plants.

Transcription factors (TFs) are a group of proteins that specifically bind to upstream sequences at the 5′ end of the genes, thereby ensuring targeted gene expression with precise spatiotemporal control. MYBs are an important family of transcription factors in higher plants [14,15]. In higher plants, there are four main types of MYB transcription factors: 1R-MYB, R2R3-MYB, 3R-MYB and 4R-MYB [16,17,18,19]. Genome-wide descriptions have been relatively detailed for model plants such as *Arabidopsis*, rice and carrot [20,21]. MYB transcription factors exhibit diverse functions in higher plants, including the regulation of various subbiological metabolic synthesis and abiotic stress responses [22,23,24,25]. MYB transcription factors have also been reported in tea plants [26,27]. *CsMYB4a* is potentially involved in the regulation of gene expression related to flavonoid biosynthesis [28]. *CsMYB2* and *CsMYB26* are two other regulatory genes involved in the biosynthesis of tea flavonoids [27].

In higher plants, MYB transcription factors are associated with the circadian rhythm [29,30,31,32,33]. The MYB transcription factors CCA1 and LHY (LATE ELONGATED HYPOCOTYL) play crucial roles in maintaining the diurnal cycle of the central oscillator in the biological rhythm system and in regulating the circadian rhythm in *Arabidopsis* [30,34]. REVEILLE1 (RVE1), a transcription factor that is similar to MYB, is homologous to LHY and CCA1. As a node in the auxin network that connects elements of the circadian rhythm, RVE1 provides a mechanistic link between two important signaling pathways [35]. Another member of the RVE family, RVE8, is an MYB transcription factor. RVE8 interacts with the transcriptional coactivators LNK1 and LNK2 to promote the expression of clock genes during nighttime [36]. Duan et al. identified a novel MYB transcription factor in rice, CMYB1, through transcriptome analysis and observed significant rhythmic expression profiles, suggesting that CMYB1 plays a crucial role in cold tolerance via circadian regulation [32]. MYB-like transcription factors LUX (LUX ARRHYTHMO), ELF3 (EARLY FLOWERING 3) and ELF4 constitute the Evening Complex (EC) [37]. In the morning, the EC was inhibited by CCA1 and LHY, and in the evening, it was inhibited by TOC1. MYB112 can directly bind to the promoter of the *LUX* gene (the core component of the central oscillator of the biological clock) and inhibit its transcription, weakening the inhibitory effect of LUX on the transcription of PIF4 and thus promoting its transcription of the *PIF4* gene [38]. Circadian rhythm gene expression and various regulatory relationships within the core oscillator are critical for maintaining the circadian rhythm. The circadian rhythm core oscillator forms a highly linked regulatory network with other circadian rhythm components, affecting a wide range of plant signal transduction and metabolic pathways [39]. 

To date, studies of MYB transcription factors have mainly focused on anthocyanin biosynthesis regulation [40,41], lignin biosynthesis regulation [42] and the abiotic stress response [43]. To the best of our knowledge, there are few reports on the circadian rhythm of tea plants. The response of MYB transcription factors to the circadian rhythm in tea plants has also not been reported. Understanding the mechanism of MYB transcription factors in regulating the circadian rhythm can enhance the adaptability and stress resistance of tea plants, change the regional restriction of tea plants and improve tea quality.

The tea plant (*Camellia sinensis* (L.) O. Kuntze) has a history of over 5000 years and boasts rich germplasm resources. As a natural, nonalcoholic beverage popular worldwide, tea plays a significant role in the prevention of cancer and cardiovascular diseases [44,45]. Tea is derived from the leaves of tea plants. As the primary organs responsible for photosynthesis and transpiration, plant leaves provide both the material and energy necessary for growth and development [46]. With the development of the tea genome and sequencing technology, omics studies of tea plants have been reported [47,48,49]. Here, the high-throughput sequencing technology DNBSEQ was used to sequence the transcriptome of the tea plant ‘Baiyeyihao’ at intervals of 4 h within one photoperiod (24 h). We identified prospective genes involved in the circadian rhythm, and differentially expressed gene transcripts with MYB transcription factor annotation information were further screened. The selected candidate genes encoding MYB transcription factors were evaluated using a heatmap to understand the response of the circadian rhythm in tea plants. Real-time fluorescence quantitative PCR was used to identify and confirm the expression profiles of circadian-rhythm-related MYB genes in tea plants. This is the first transcriptome analysis of circadian rhythm transcription factors in tea plants. These results also provide a reference for further research on the roles of MYB transcription factors in the circadian rhythm of higher plants.

## 2. Results

### 2.1. Sequencing Quality Analysis 

Through transcriptome sequencing analysis of tea leaves at a photoperiod interval of 4 h (0 h, 4 h, 8 h, 12 h, 16 h, 20 h), 10.42~10.45 Gb of effective data was obtained. The values of Q20 (error rate less than 1%) were above 96.21%, and the values of Q30 (error rate less than 1%) were above 91.00%. The percentages of GC contents were 45.046%, 44.611%, 44.659%, 44.157%, 44.306% and 44.529%, respectively (Table 1). 

### 2.2. Analysis of Gene Transcripts 

**Figure 1:**
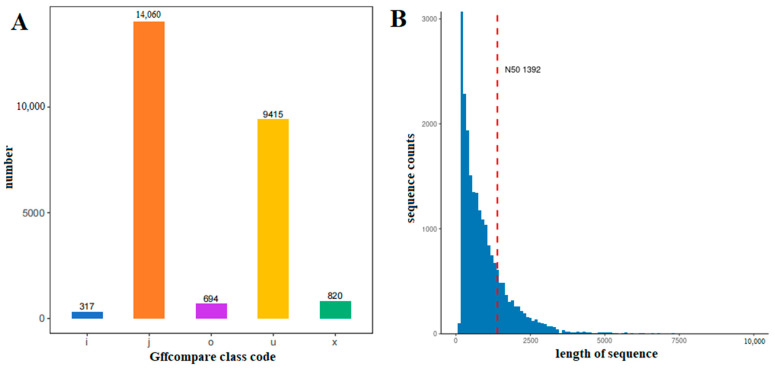
Quantity and differential gene transcript (DET) length distribution of new transcript types in *C. sinensis* within a photoperiod. (**A**): Quantity distribution of new transcript types in the sample; ‘o’: other overlapped parts of the same chain with reference exons; ‘j’: multiple exons with at least one match; ‘x’: exon overlap on the antichain; ‘i’: completely contained in the intron of the reference transcript; ‘u’: unknown new transcripts. (**B**): Differential gene transcript length distribution of new transcripts. The abscissa indicates the length of reads. The ordinate indicates the number of reads in that length range, where the red dotted line indicates the length of N50.

Adaptor sequences, duplicated sequences and low-quality reads were excluded. The filtered data were qualitatively controlled using fastqc, and the StringTie software (Version: 2.1.4; Parameter: default) was used to assemble the transcripts. In total, 25,306 transcripts were obtained. Among them, ‘o’ is other overlapped parts of the same chain with reference exons, with a total of 694; ‘j’ is for multiple exons with at least one match, of which there are 14,060; ‘x’ is exon overlap on the antichain; ‘i’ is completely contained in the intron of the reference transcript; and ‘u’ is 9415 unknown new transcripts. TransDecoder was used to predict the encoding region of a new transcript. The results showed that the length of the unigenes was mainly between 100 and 1500 bp, and the length of the N50 was 1392 bp (Figure 1).

### 2.3. Functional Annotation and Categorization

To obtain functional information on the transcripts, functional annotations were drawn up for transcripts from seven databases: Nr (NCBI non-redundant) [50], Pfam [51], Uniprot [52], KEGG (Kyoto Encyclopedia of Genes and Genomes) [53], GO (Gene Ontology) [54], COG (Clusters of Orthologous Groups) [55] and PATHWAY [53]. A total of 19,872 unigenes were annotated in the Nr database, accounting for 78.53%. A total of 19,641 unigenes were annotated in the Uniport database, accounting for 77.61%. A total of 48 unigenes were annotated in the KOG database, accounting for 0.19%. A total of 14,615 unigenes were annotated in the GO database, accounting for 57.75%. A total of 5779 unigenes were annotated in the KEGG database, accounting for 22.84% (Supplementary Table S1).

**Figure 2:**
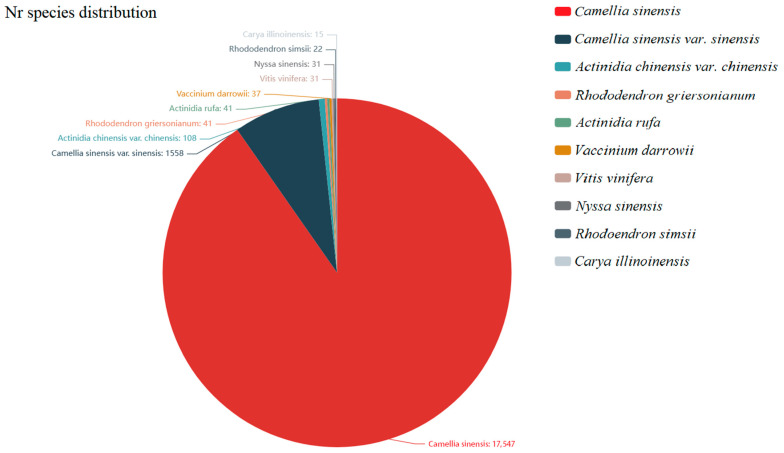
Nr annotated species distribution map in *C. sinensis*. The top 10 species with the most comparisons were counted, and the numbers represent the numbers of different species in the Nr database, while different colors represent different species. The species distribution of BLAST hits for each unigene was determined with a cut-off of 1 × 10^−5^.

The 25,306 assembled unigenes were compared with the related databases. Among these, 19,872 unigenes were annotated in the Nonredundant Collection of Nucleotide Sequences (nr). According to the comparative annotation results of the Nr database with *Camellia sinensis* as a reference, the top 10 species with the most comparisons were determined to be as follows: *Camellia sinensis* (17,547), *C. sinensis* var. *sinensis* (1558), *Aactinidia chinensis* var. *chinensis* (108), *Rhododendron griersonianum* (41), *Actinidia rufa* (41), *Vaccinium darrowii* (37), *Vitis vinifera* (31), *Nyssa sinensis* (31), *Rhodoendron simsii* (22) and *Carya illinoinensis* (15) (Figure 2).

### 2.4. GO Analysis 

**Figure 3:**
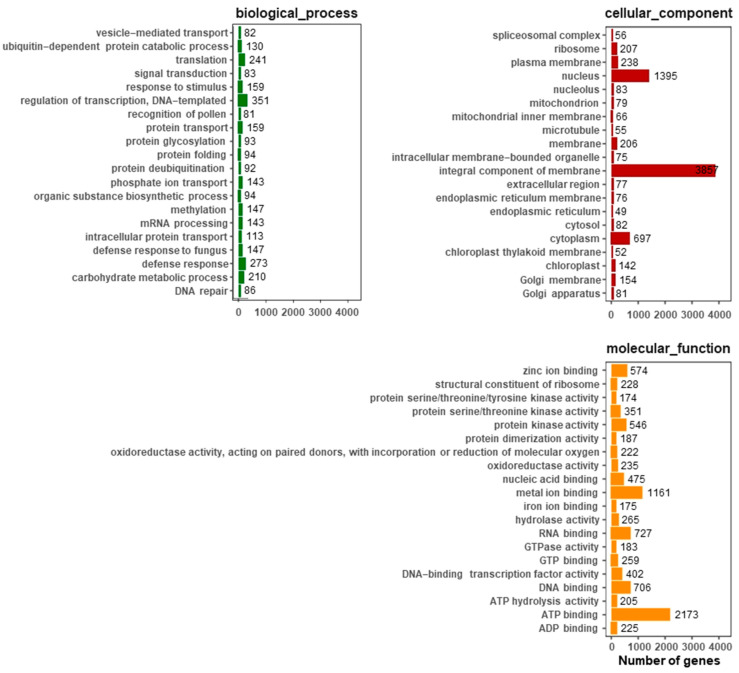
GO annotation of functional genes in *C. sinensis*. We selected the top 20 most annotated GOslim under each classification, and each unigene was classified into at least one GO term. All unigenes were grouped into three categories: molecular function, cellular component and biological process.

Gene Ontology (GO) is an international standard classification system of gene function. The GO database was used to classify the annotated tea transcriptome data. A total of 14,615 unigenes of *C. sinensis* had Gene Ontology (GO) annotations. GO describes gene function from three aspects: cellular component (CC), molecular function (MF) and biological process (BP). The top 20 most common GOslim secondary categories in each category were selected for plotting (Figure 3). Among them, transcription regulation & DNA—templated (351), defense response (273) and translation (241) involved the largest number of differentially expressed gene transcripts (DETs). Among the cell components, the integral components of the membrane (3857), nucleus (1395) and cytoplasm (697) were involved in the most DETs. The most common terms in molecular functions were ATP binding (2173), metal ion binding (1161) and RNA binding (727).

### 2.5. KEGG Classification 

**Figure 4:**
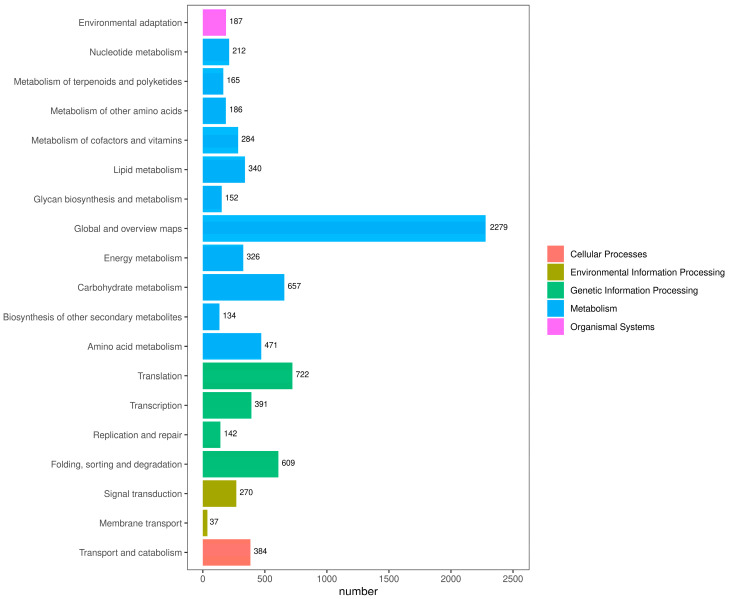
KEGG metabolism pathway categories in *C. sinensis*. Different colors represent the five classes involved in the KEGG metabolic pathway, and numbers represent the numbers of DETs in different classes.

After KEGG annotation of transcript sequences, they were classified according to the KEGG metabolic pathways in which they participated, and the results are shown in Figure 4. A total of 10,443 DETs were annotated. This classification is divided into five levels: cellular processes, environmental information processing, genetic information processing, metabolism and organismal systems. The largest level of these was metabolism, which had 11 sublevels. Among the 11 sublevel metabolic pathways were global and overview maps (2279), carbohydrate metabolism (657) and amino acid metabolism (471). In the processing of genetic information, translation (n = 722) and folding, sorting and degradation (n = 609) occupied large proportions. 

### 2.6. Differential Gene Transcript Analysis

**Figure 5:**
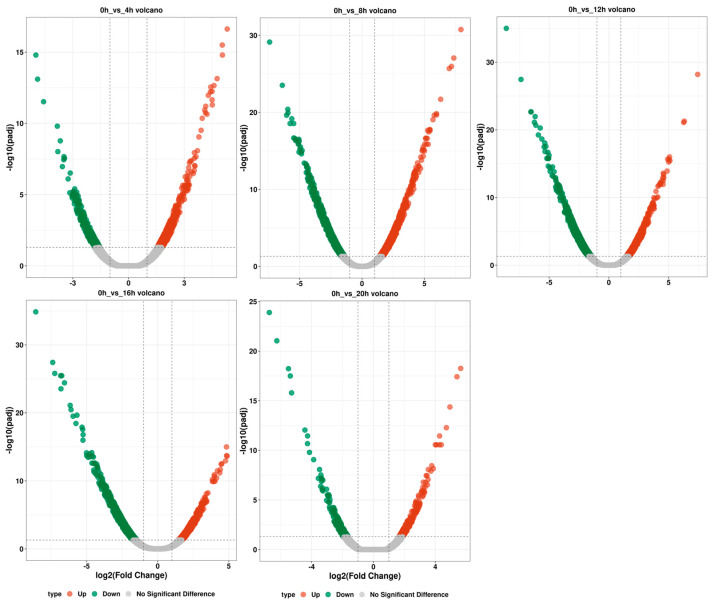
Volcano map of differentially expressed gene transcripts. Each point represents a transcript, and the horizontal coordinate is logFC, which represents the base −2 pair value of the expression difference multiple of a certain transcript in the two samples. The ordinate represents the base 10 pair value of PValue (too few differential transcripts) or error detection rate FDR multiplied by −1.

In our differential expression analysis, the expression levels of the gene transcripts in each sample were obtained through expression quantitation of read count data. A total of 772, 2411, 1731, 1434 and 393 differentially expressed gene transcripts were identified in “0 h_vs._4 h”, “0 h_vs._8 h”, “0 h_vs._12 h”, “0 h_vs._16 h” and “0 h_vs._20 h.” In “0 h_vs._4 h”, a total of 469 differentially expressed gene transcripts were upregulated and 303 were downregulated. In “0 h_vs._8 h”, a total of 1215 differentially expressed gene transcripts were up- and 1196 downregulated. In “0 h_vs._12 h”, a total of 697 differentially expressed gene transcripts were up- and 1034 downregulated. In “0 h_vs._16 h”, a total of 532 differentially expressed gene transcripts were up- and 902 downregulated. In “0 h_vs._20 h”, a total of 235 differentially expressed gene transcripts were up- and 158 downregulated (Figure 5).

### 2.7. Differential Expression of Gene Transcripts Encoding Transcription Factors

**Figure 6:**
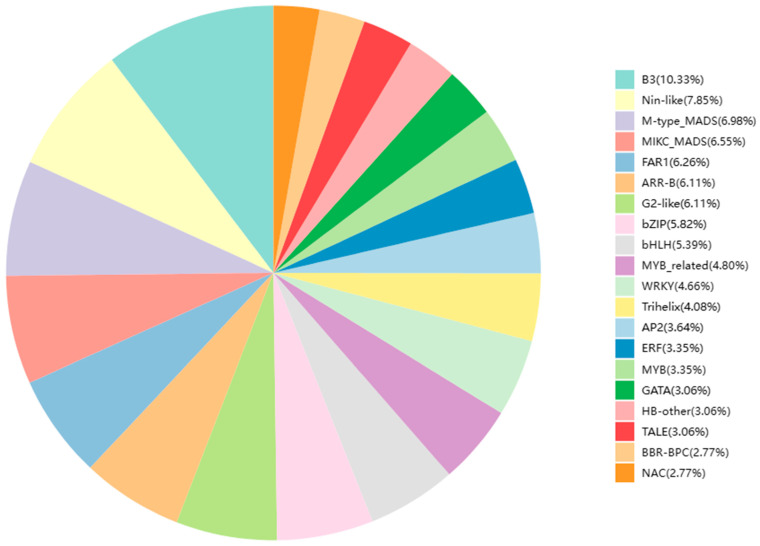
Analysis of transcription factor families in *C. sinensis*. Different colors represent different families of transcription factors, and the numbers in brackets indicate the proportions to the total differential transcription factors.

DETs encoding transcription factors were predicted using PlantTFDB [56]. The top 20 transcription factor families with differential expression were selected for plotting, and the proportion statistics are shown in Figure 6. A total of 894 DETs encoding transcription factors were detected, the most abundant of which were the B3 (71) and Nin-like (54) transcription factor families. Twenty-three MYB transcription factors and thirty-three MYB-related transcription factors were identified, accounting for 3.35% and 4.80% of the total differentially expressed transcription factors (894), respectively. According to the results of transcription factor annotation, DETs with transcription factor annotation information were further screened, and duplicate transcription factors were removed. A total of 22 transcripts of the MYB transcription factors were obtained for subsequent analysis. We identified one upregulated and four downregulated unigenes in “0 h_vs._4 h”; two upregulated and twelve downregulated unigenes in “0 h_vs._8 h”; one upregulated and eight downregulated unigenes in “0 h_vs._12 h”; one upregulated and eight downregulated unigenes in “0 h_vs._16 h”; and one upregulated and zero downregulated unigenes in “0 h_vs._20 h.”

### 2.8. Distribution of the DETs Encoding MYB Transcription Factors

**Figure 7:**
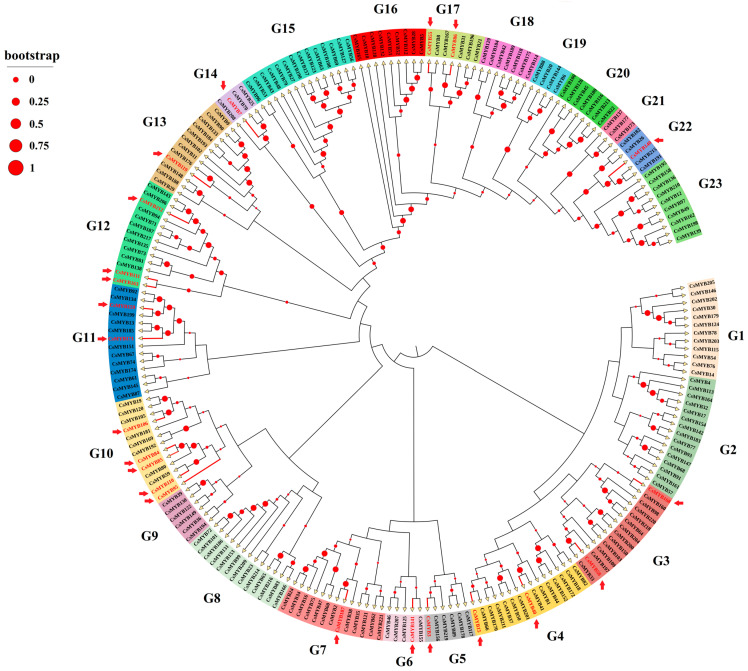
Evolutionary tree analysis of MYB transcription factor family in *C. sinensis*. Arrows represent 22 differentially expressed MYB transcription factors associated with circadian rhythm. Different colors indicate different subfamilies, and red circles indicate similarity.

A total of 222 MYB transcription factors were retrieved from the TPIA database. Multiple sequence alignment was performed on those, and an NJ phylogenetic tree was generated based on the alignment results (Figure 7). The results showed that tea plant MYB members can be divided into 23 subfamilies, named Groups 1 to 23. The 22 selected DETs encoding MYB and MYB-related transcription factors related to the circadian rhythm of tea plants were distributed among the different subfamilies. The numbers of DET MYB transcription factors belonging to the G10, G11 and G12 groups were higher than those in the other groups. Among them, the G10 group had the highest number of transcripts (a total of five) associated with the circadian rhythm. In the G1, G2, G8, G9, G15, G16, G18, G19, G20, G21 and G23 groups, no member was associated with the circadian rhythm.

### 2.9. Expression Analysis of MYB Transcription Factors in the Circadian Rhythm of Tea Plants

**Figure 8:**
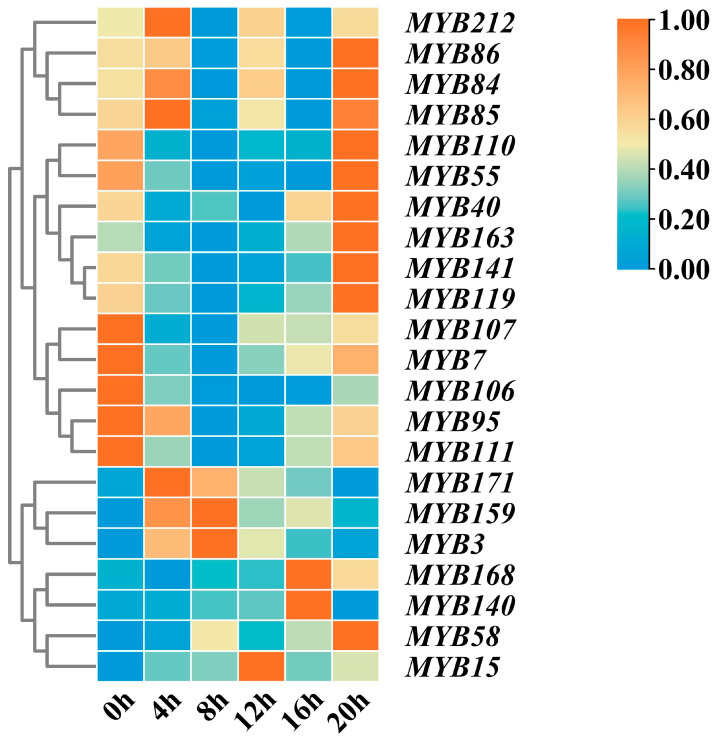
Heatmap of expression profiles of MYB transcription factors within a photoperiod. Color scores were normalized by the counts of RPKM values. Red represents high expression. Blue represents low expression.

In studying the expression patterns of the 22 MYB transcription factors in the circadian rhythm of tea plants, heatmap analysis showed the expression levels of the gene transcripts within a photoperiod (Figure 8). The heatmap showed that the expression levels of *MYB3*, *MYB159* and *MYB171* were high during the day and low at night, and the process from 0 h treatment showed an obvious rise to fall. The expression levels of *MYB15*, *MYB58*, *MYB140* and *MYB168* were high at night and low during the day, showing a process of decline to rise. The expression levels of *MYB84*, *MYB85*, *MYB86* and *MYB212* were the highest at 0 h and then began to decline, rise again, fall again and rise again at night, then reached a peak, showing a zigzag pattern. The expression levels of *MYB7*, *MYB95*, *MYB106* and *MYB107* were the highest at 0 h, then began to decline and then began to increase after 20 h treatment. The daytime period is 0 h–12 h (9:00–21:00) on the horizontal coordinate, and the nighttime period is 12 h–20 h (21:00–5:00) on the horizontal coordinate. 

The 22 MYB gene transcripts showed differential responses in terms of tea plant circadian rhythm based on the results of the RPKM method (Table S3). The expression levels of *MYB3*, *MYB7*, *MYB15*, *MYB40*, *MYB55*, *MYB58*, *MYB84*, *MYB85*, *MYB86*, *MYB95* (*RVE8*), *MYB106* (*LHY*), *MYB107*, *MYB110*, *MYB111*, *MYB119*, *MYB140*, *MYB141*, *MYB159*, *MYB163* (*RVE1*), *MYB168*, *MYB171* and *MYB212* were measured and analyzed using RT-qPCR. The results showed that the gene expression profiles of the 22 MYB transcription factors can be divided into four patterns according to photoperiod: low expression level in a photoperiod, high expression level during the day and low expression level at night, low expression level during the day and high expression level at night, and irregularity. In general, the expression levels of the 22 DETs encoding MYB transcription factors in the tea plant circadian rhythm were consistent with the trend of the transcriptome sequencing results.

### 2.10. The Expression Profiles of the MYB Gene Transcripts

#### 2.10.1. High Expression Level in the Day and Low Expression Level at Night

**Figure 9:**
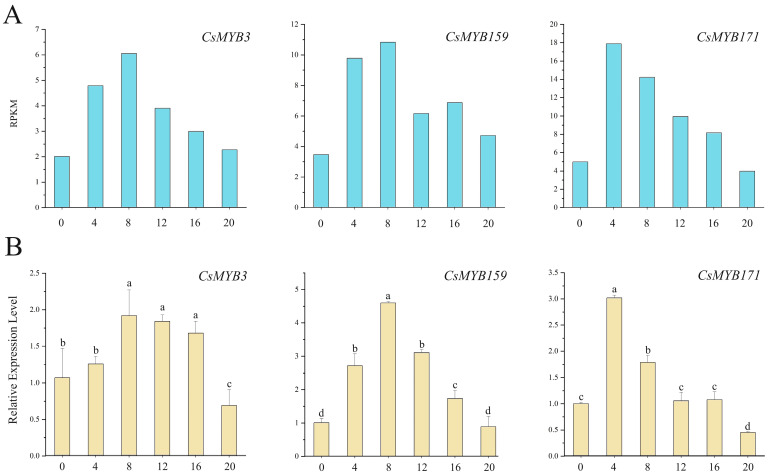
The expression levels of CsMYB gene transcripts in *C. sinensis* within a photoperiod, with profiles that are high in the day and low at night. (**A**): Reads per kilobase per million mapped reads. (**B**): Real-time PCR. The standard deviation (SD) is represented by the error bars. Different lowercase letters indicate significant differences at the 0.05 level.

**Figure 10:**
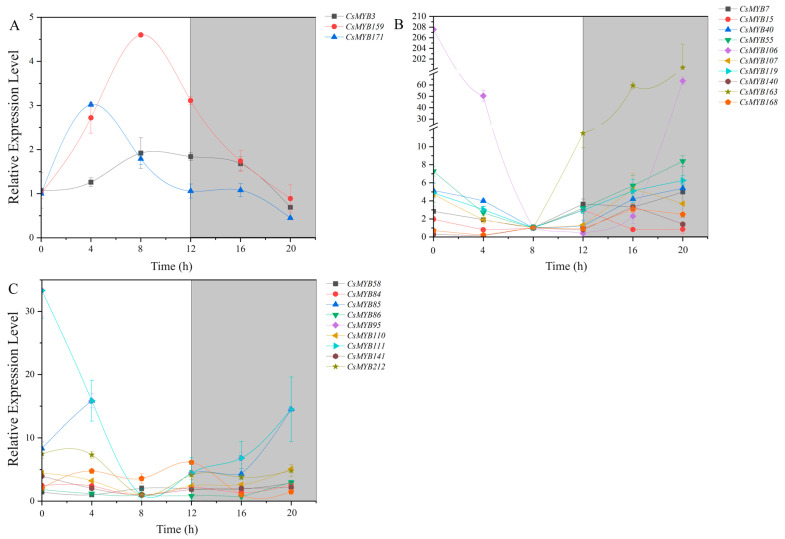
Dynamic behavior of MYB transcription factor in *C. sinensis* within a photoperiod. (**A**). Dynamic behavior of high expression level during the day and low expression level at night. (**B**). Dynamic behavior of low expression level during the day and high expression level at night. (**C**). Dynamic behavior of the expression level irregularity and little change. The gray bands denote dark, and the white bands represent light.

When studying the RPKM (Figure 9A) and quantitative RT-PCR (Figure 9B) results, it was found that the expression levels of *MYB3*, *MYB159* and *MYB171* gene transcripts were high in the day and low at night. The *MYB3* and *MYB159* gene transcripts reached a peak value at 8 h and the lowest value at 20 h. The expression levels of *MYB3* and *MYB159* at the peak value were 2.78 and 5.17 times higher than that of the lowest value, respectively. The *MYB171* transcript reached its peak value at 4 h and reached its lowest value at 20 h, which was 6.71 times higher. The expression level of *MYB171* at the peak value was 6.71 times higher than that at the lowest value. The transcript levels of *MYB3*, *MYB159* and *MYB171* first increased and then decreased, which had a certain regularity (Figure 10A).

#### 2.10.2. Low Expression Level in the Day and High Expression Level at Night

**Figure 11:**
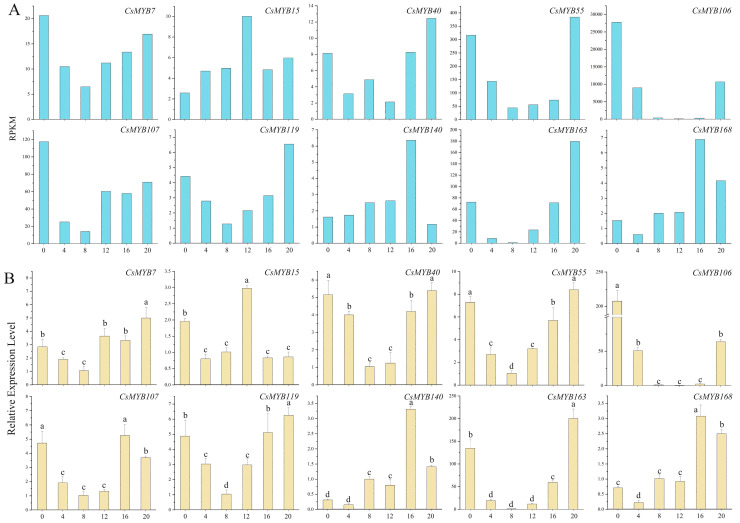
The expression levels of CsMYB gene transcripts in *C. sinensis* within a photoperiod, with profiles that are low in the day and high at night. (**A**): Reads per kilobase per million mapped reads. (**B**): Real-time PCR. The standard deviation (SD) is represented by the error bars; different lowercase letters indicate significant differences at the 0.05 level.

The expression levels of *MYB7*, *MYB15*, *MYB40*, *MYB55*, *MYB106* (*LHY*), *MYB107*, *MYB119*, *MYB140*, *MYB163* (*RVE1*) and *MYB168* were high at night and low in the day based on the results of the RPKM method (Figure 11A). *MYB106* (*LHY*) and *MYB163* (*RVE1*) are the core genes of the circadian rhythm. The expression level of the LHY transcript was the highest at 0 h and 20 h, when the expression level was up to 207.57 and 63.57 times that at 8 h, respectively. The expression level of the RVE1 transcript increased gradually from 12 h to 20 h, while the expression level was low during the day. The expression level of the RVE1 transcript decreased gradually from 0 h to 8 h. The peak expression levels were more than 200 times higher than the trough levels (Figure 11B). The expression levels of these MYB gene transcripts showed a trend of first decreasing and then increasing (Figure 10B). 

#### 2.10.3. The Expression Level Irregularity and Little Change

**Figure 12:**
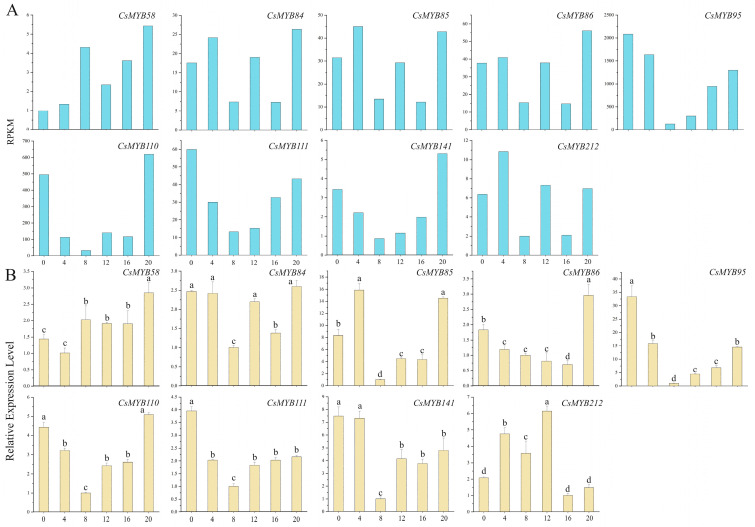
The expression levels of CsMYB gene transcripts with irregular expression profiles in *C. sinensis* within a photoperiod. (**A**): Reads per kilobase per million mapped reads. (**B**): Real-time PCR. The standard deviation (SD) is represented by the error bars. Different lowercase letters indicate significant differences at the 0.05 level.

A total of 222 MYB transcription factor transcripts were found in the tea plant database. Through transcriptome identification and screening, it was estimated that the expression levels of the other 200 MYB gene transcripts showed little change during the photoperiod (24 h) (Figure 10C). The genes of *MYB84*, *MYB85*, *MYB86* and *MYB141* were highly expressed during both the day and night (Figure 12A,B).

## 3. Discussion

Tea plants are typically woody crops with rich genetic diversity during long-term natural growth [57]. In tea plants, the accumulation of secondary metabolites is inconsistent during the day and night and is affected by various factors, including the circadian rhythm [58]. At present, research on the circadian rhythm is mainly concentrated on *Arabidopsis* model plants, which are also the most in-depth plants in terms of photoperiod regulatory mechanisms [5,6]. Research of the circadian rhythm of horticultural plants has just started, mainly involving tomatoes [6], garlic [59], cabbage [60], etc.

At present, 58 transcription factor families have been identified in plants, among which AP2/ERF, MYB, GRAS, WRKY and NAC are the transcription factor families studied in-depth [61,62]. The MYB family of genes exhibits differential expression patterns in plant tissues and performs specific physiological regulatory functions [63]. The MYB family is one of the largest families of transcription factors in plants and plays a crucial role in the regulation of plant biochemical and physiological processes. Syntenic analysis demonstrated that tandem duplication contributed to its evolution. It has also been shown that the main drivers of peanut *R2R3*-*MYB* gene amplification are polyploidy and tandem and single repetition [64,65]. Chen et al. identified a total of 122 CsR2R3-MYB genes based on the chromosome-level genome of tea plants. *CsMYB12*, *CsMYB17*, *CsMYB25* and *CsMYB47* genes exhibited high expression in the tea plant leaves under PEG stress, but low expression under cold stress. At the same time, they found that *CsMYB17* enhanced anthocyanin accumulation in tea plants [43]. *CsMYB42* played a crucial role in activating the expression of CsGS1c and might be involved in the biosynthesis of theanine in albino tea leaves [66]. Li et al. predicted that *CsMYB8* and *CsMYB99* were involved in the biosynthesis of flavonoids (including catechins, anthocyanins and flavanols), while *CsMYB9* and *CsMYB49* were associated with theanine synthesis, as well as the MYB TFs that were likely involved in hormone-signaling-mediated environmental stress and defense responses [58]. A total of 222 transcripts of the MYB transcription factor are found in tea plants [57]. MYB transcription factors also play key roles in the regulation of the growth and development of tea plants [16]. In this study, information about MYB transcription factors was based on data provided by Li et al. [58]. According to the TPIA database, MYB transcription factors were systematically named CsMYB1–CsMYB221. The corresponding MYB transcription factor names were obtained through BLAST comparison (Table S4). RT-qPCR primers were designed using Primer Premier 6.0 software.

Nearly one-third of *Arabidopsis* genes are regulated by the circadian rhythm, with their expression peaking at different times of the photoperiod [67,68]. In plants, the proteins encoded by *CCA1* and *LHY* are important components in maintaining the diurnal cycle in the central oscillator of the biological clock system of higher plants and regulating the circadian rhythm [30,34]. The *LCL5* (*RVE8*) and *CCA1* gene sequences were homologous. However, these two transcription factors have opposite regulatory effects on the circadian rhythm expression of the *Arabidopsis TOC1* gene [69]. *CCA1* and its closely related homolog *LHY* encode a single MYB transcription factor that regulates the circadian rhythm of genes expressed in the morning and evening. The morning cycle is composed of *CCA1*, *LHY*, *PRR7* (*PSEUDO-RESPONSE REGULATOR 7*) and *PRR9* (*PSEUDO-RESPONSE REGULATOR 9*), in which *CCA1* and *LHY* can promote the transcription and expression of *PRR7* and *PRR9*. However, *PRR7* and *PRR9* can reverse the inhibition of *CCA1* and *LHY* [70]. The abundance of *CCA1* and *LHY* mRNA transcripts oscillates rhythmically, with peak expression at dawn and trough expression at dusk [71,72]. Similar to the expression patterns of *CCA1* and *LHY*, the expression of *RVE1*, *RVE2* and *RVE7* is clock-regulated, with peak transcriptional abundance near subjective dawn, suggesting that these genes may play an important role in clock regulation during the morning phase [73], which is also consistent with the results of this study. In addition, CCA1 and LHY play crucial roles in flowering. Flowering marks the transition from vegetative to reproductive growth, and proper flowering timing is critical for reproduction [74]. CCA1 and LHY participate in the regulation of photoperiodic flowering by regulating the GI-CO-FT pathway [75].

The MYB transcription factor is closely related to the circadian rhythm. By screening the DETs with transcription factor annotation information, a total of 22 MYB and MYB-related transcription factors related to the circadian rhythm of tea plants were obtained, including the typical circadian rhythm genes *LHY* (*MYB106*), *RVE1* (*MYB163*) and *RVE8* (*MYB95*). *REVEILLE1* (*RVE1*), which is homologous to *LHY* and *CCA1*, is an MYB-like transcription factor. Another member of the RVE family, *RVE8*, is an MYB transcription factor. RVE8 interacts with the transcriptional coactivators LNK1 and LNK2 to promote the expression of night clock genes [36]. RVE1 regulates auxin biosynthetic genes to connect the circadian clock and auxin pathways [35]. RVE8 regulates anthocyanin biosynthesis by promoting the expression of anthocyanin genes [76]. *GmMYB133* is a soybean *RVE* homologous gene involved in the biosynthesis of soybean isoflavones [77]. Transient overexpression of *PbRVE1b* can increase the anthocyanin content in birch peels [78]. These results support the importance of the RVE protein in plant development. All *PbRVE* genes in *Pyrus bretschneideri* leaves under a light/dark cycle exhibited a circadian rhythm. The four *PbRVE* genes also exhibited robust rhythms under constant light conditions [79]. In plants, *Arabidopsis* and *Oenanthe javanica* grow under various photoperiodic conditions, and the expression of the clock gene *Lhcb* always decreases from peak levels before dusk and increases from trough levels before dawn. This also suggests that the significance of circadian clock adaptation may be partly due to its regulation of light responses to photosynthesis and photoprotection mechanisms [80,81,82]. The circadian oscillator may contain multiple circuits that mediate positive and negative feedback, respectively. Interlocked feedback loops have been described in the circadian rhythm in Drosophila, tomatoes, fungi, etc. [7,83,84]. 

*PbRVE* expression patterns differed in different tissues of *P. bretschneideri*, suggesting that *PbRVE*s may have a potential function in plant development. *PbLHY* and *PbRVE8* are highly expressed in leaves, which is consistent with the previously reported cognates of poplar [85] and rice [86]. In addition, transcripts of *RVE* genes, such as *PbRVE3b* and *PbREVE6b*, show higher levels in the root of *P. bretschneideri*. Recently, transcriptome analysis of walnuts and alfalfa revealed that *RVE* genes may be involved in nitrogen metabolic pathways. 

We measured expression patterns in different tissues of tea plants and further explored the function of MYB transcription factors in the following directions. Since the circadian rhythm in tea plants has not yet been reported, we are still in the preliminary stage of research. Changing the circadian rhythm of the tea plants and determining whether the growth state of the tea plant can be changed is also a good research direction.

## 4. Materials and Methods

### 4.1. Plant Materials

Cutting seedlings of two-year-old ‘Baiyeyihao’ (*C. sinensis* cv. Baiyeyihao) were planted in the growth chamber at the Tea Science Research Institute of Nanjing Agricultural University (Nanjing, China, 188.84° E, 32.04° N). Tea seedlings were planted in sandy loam with good drainage, an organic matter content over 1–2% and a pH value of 6.0. Tea seedlings with healthy growth were selected and cultured in a light incubator (temperature 25 ± 2 °C, photocycle 12 h light/12 h dark, light intensity 240 μmol·m^−2^·s^−1^, humidity 70 ± 10%) for one week. Plants were adjusted daily to ensure well-rounded lighting and watered daily early and late in the day. According to literature reports, the circadian rhythm time scheme was experimentally assessed with 4 h intervals [87]. A photocycle of 12 h/12 h is the normal growth time of the plant. Therefore, in order to better understand the influence of the circadian rhythm on the tea plants, we divided 24 h into 6 segments. At 9:00, the illumination began (the initial time was denoted as 0 h) and the first sample was taken. Then, healthy tea seedlings were selected at 4, 8, 12, 16 and 20 h, respectively. Horizontal coordinates 0 h, 4 h and 8 h belonged to the daytime period (9:00~17:00), while 12 h, 16 h and 20 h belonged to the night period (21:00~5:00). An individual sample containing one bud and two leaves was picked from one tea plant, quickly frozen with liquid nitrogen and stored at −80 °C. Three biological replicates were performed for each sample.

### 4.2. RNA Extraction

Extraction of total RNA from tea leaves was performed using a reference RNA extraction kit (RNA simple total RNA Kit, Beijing Tiangen Company, Beijing, China). The concentration of RNA samples was determined using micro ultraviolet detector Nanodrop ND-1000 (Shanghai Spectrometer, Shanghai, China). The RNA quality was detected via 1.2% agarose gel electrophoresis.

### 4.3. cDNA Library Construction and High-Throughput Sequencing 

Six groups of RNA samples were sent to Bena Technology Co., Ltd. (Wuhan, China) for construction and sequencing of the cDNA library. mRNA was enriched using oligo (dT) magnetic beads. Interrupt reagent was added to the enriched mRNA to fragment the mRNA. The interrupted mRNA was synthesized into one-strand cDNA and two-strand cDNA according to the corresponding procedure. After terminal repair, adding an A tail, adding a sequencing joint, purification, PCR amplification and product cyclization were among the steps were followed to complete the preparation of the library. We did so using the MGIEasy RNA Library Prep Kit (Huada intelligent manufacturing Technology Co., Ltd., Shenzhen, China). After qualified library detection, the high-throughput sequencing platform DNBSEQ was used for sequencing.

### 4.4. RNA-Seq Data and Enrichment Analysis of Differentially Expressed Transcripts

The transcription factors were predicted through the database, and the top 20 transcription factors were selected for analysis. In this study, we focused on MYB transcription factors involved in the response to the circadian rhythm. According to the transcription factor annotation results, after removing duplicate transcription factors, 22 transcripts of the MYB transcription factor were obtained. Since the original sequencing data may contain low-quality sequences, joint sequences, etc., in order to ensure the reliability of the information analysis results, the original sequencing data should be filtered to obtain clean reads. After quality control in FastQC (version: 0.11.9; Parameter: default) [88], the transcriptome samples were trimmed using Trim Galore [89]. The sequenced reads were mapped to the reference genome of tea plants using HISAT2 [90]. The reads for each gene were counted using Subread-featureCounts with default parameters [91]. The number of reads from each sample to each transcript was obtained using RSEM (RNA-Seq by Expectation Maximization) and converted using FPKM (Fragments Per Kilobase per Million bases) [92]. Paired-end reads from the same fragment were counted as one fragment to obtain the gene and transcript expression levels. Furthermore, differentially expressed gene transcripts (DETs) were identified using the edgeR package with FDR < 0.05 and |logFC| > 1.5. If the number of significantly different transcripts is too small, the screening threshold is *p*-value < 0.05 and |logFC| > 1.5. FDR values were used for significant difference filtering in this analysis [93]. This code is provided in Supplementary File S2.

### 4.5. Functional Annotation

In order to obtain comprehensive functional information of the new transcripts, the obtained Unigene sequences were BLAST analyzed against Nr, SwissProt, GO, KEGG, COG and other databases for protein functional annotation and classification of tea plant genes. KEGG annotations were associated with KEGG ORTHOLOGY and PATHWAY using KOBAS (version: 3.0). The Uniprot [52] database records the correspondence between each protein family and functional nodes in Gene Ontology [54]. This system predicts the biological functions of the protein sequence encoded by a transcript.

### 4.6. Differential Genes Were Verified Using qRT-PCR 

Real-time quantitative PCR (qRT-PCR) was used to detect the expression levels of the 22 selected MYB gene transcripts related to the circadian rhythm in tea plants. Detection primers were designed using Primer Premier 5.0 software. The SYBR Premix Ex Taq kit (TaKaRa, Dalian, China) was used for qRT-PCR on the Bio-Rad IQ5 fluorescence quantitative PCR platform. The total RNA extracted from tea leaves was reverse-transcribed into cDNA using a reverse transcription kit (TaKaRa Biotech Co., Ltd., Dalian, China). The cDNA was diluted 18-fold with nuclease-free deionized water, and 2 μL of each sample was extracted as a template added to the reaction mixture (20 μL). Each cDNA required 1 μg of RNA. In this study, the *CsGAPDH* gene was selected as the internal reference gene in qRT-PCR analysis, and the amplification primers were CsGAPDH-F and CsGAPDH-R [48]. It has been reported that the stability of the *GAPDH* gene is high in *Coffea arabica* [94]. Wu et al. showed that *CsGAPDH* has a reasonable expression abundance (19 < *Cq* < 30) and can be used in the calculation of gene standardization in tea plants [48]. The cDNA of ‘Baiyeyihao’ was selected at intervals of 4 h within one photoperiod (24 h). The systems used for amplification were 20 μL: 10 μL SYBR Green I mix, 0.4 μL forward and reverse fluorescent quantitative primers, 2.0 μL cDNA, 7.2 μL ddH_2_O [95]. The amplification program was set at 95 °C for 5 min, denatured at 95 °C for 10 s, annealed at 54 °C for 30 s and extended at 65 °C for 15 s, for a total of 40 cycles. The final concentration of primers in the reaction mix was 0.2 μM. Three biological replicates were performed, and 2^−ΔΔCT^ was used to calculate relative gene expression levels [96]. Primers for RT-qPCR of MYB gene transcripts and the internal reference gene are listed in Table S2.

### 4.7. Data Processing and Analysis 

Microsoft Excel 2019 software was used for data sorting. The significance of differences was analyzed using IBM SPSS Statistics 25.0, and Origin 8.0 software was used to complete the graph production. Heat maps were perpared using TBtools software (Version: 1.045) [97].

## 5. Conclusions

In this study, to explore the differential response of MYB transcription factors to the circadian rhythm of tea plants, the transcriptome of the tea plant ‘Baiyeyihao’ was sequenced at intervals of 4 h within one photoperiod (24 h). Then, 22 DETs encoding MYB and MYB-related transcription factors were identified, including three core oscillator genes (*LHY*, *RVE1* and *RVE8*). There are four types of circadian expression patterns of MYB transcription factors within a photoperiod in tea plants. Among them, the expression levels of the core oscillator genes *LHY* and *RVE1* were high at night and low during the day. The regulatory mechanism of the tea plant circadian rhythm is complex and is affected by various environmental factors. This study provides potential basic data for further research on the relevant regulatory mechanism of MYB gene transcripts in response to the circadian rhythm in tea plants.

## Figures and Tables

**Table 1:** Tea plant sequencing data volume statistics in a photoperiod.

Sample	Clean Reads (n)	Clean Bases (bp)	Q20 Rate/%	Q30 Rate/%	GC Content/%
0 h	70,014,514	10,426,133,452	97.201	91.627	45.046
4 h	70,039,110	10,436,107,844	96.278	91.847	44.611
8 h	70,054,394	10,441,015,266	96.206	91.676	44.659
12 h	70,005,768	10,434,845,980	97.420	92.273	44.157
16 h	70,049,578	10,445,672,732	97.170	91.545	44.306
20 h	70,017,578	10,420,595,674	96.997	91.001	44.529

## Data Availability

Raw RNA-Seq reads can be found in the Genome Sequence Archive database (https://bigd.big.ac.cn/gsa/browse/CRA013441, accessed on 14 November 2023) with Bio Project ID CRA013441; the expression data (FPKM) are on Dryad (https://datadryad.org/stash/share/IJVErepIxRijiJis_wFRaBaIsS8BtDaT1oM5wy3yQX0, accessed on 22 December 2023).

## Supplementary Materials

The following supporting information can be downloaded at: https://www.mdpi.com/article/10.3390/ijms25010657/s1.
